# Supplementary material for: Association of Prolonged Fluoroscopy Time with Procedural Success of Percutaneous Coronary Intervention for Stable Coronary Artery Disease with and without Chronic Total Occlusion
Source: J Clin Med. 2021 Apr 3;10(7):1486. doi: 10.3390/jcm10071486 (PMC8038393; doi:10.3390/jcm10071486)
Supplement: Supplementary file 1 [file jcm-10-01486-s001.pdf]

Supplement

Supplementary Figure 1. Receiver operating characteristic (ROC) curve analysis of fluoroscopy time (minutes) and procedural success of performed percutaneous coronary interventions overall (**Panel A**), non-CTO PCI (**Panel B**) and CTO PCI (**Panel C**).

AUC, area under the curve, CTO, chronic total occlusion; PCI, percutaneous coronary intervention.

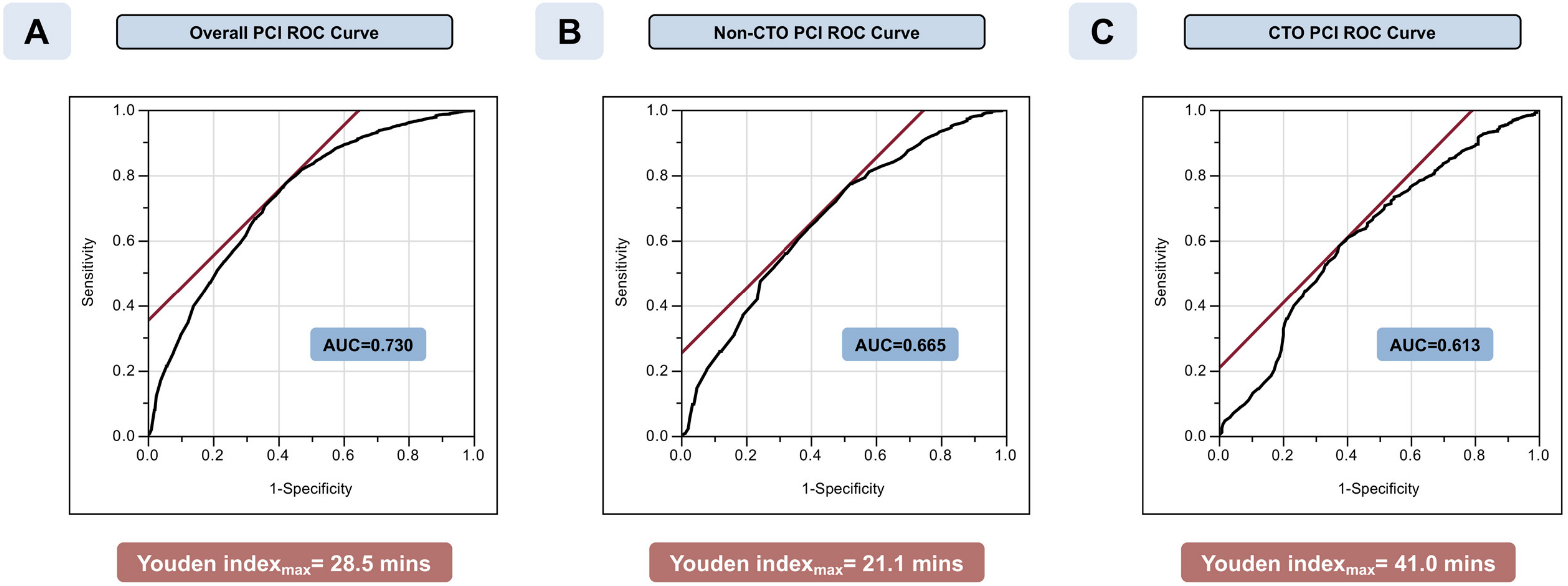

Supplementary Table 1. Sensitivity logistic regression between chronic occlusive (CTO) percutaneous coronary intervention (PCI), fluoroscopy time (both as independent variables) and procedural failure (as dependent variable) by applying the Society of Cardiovascular Angiography and Interventions (SCAI) myocardial infarction (MI) definition.

**Variable**	**Procedural failure (SCAI MI)**		
	**OR**	**CI 95%**	**P value**
CTO PCI (vs. non-CTO PCI)	2.16	1.81-2.58	**<0.001**
Fluoroscopy time (mins)	1.03	1.02-1.03	**<0.001**
CTO PCI * Fluoroscopy time (mins)	**-**	**-**	**<0.001**

Supplementary Table 2. Baseline characteristics of chronic occlusive (CTO) and non-occlusive (non-CTO) percutaneous coronary intervention (PCI) with and without procedural success.

**Variable**	**CTO PCI**		**P value**	**Non-CTO PCI**		**P value**
	**Success (n=2408)**	**Failure (n=312)**		**Success (n=9592)**	**Failure (n=226)**	
Age (years)	67.9 ± 10.0	65.2 ± 10.4	**<0.001**	70.3 ± 10.1	72.1 ± 10.1	**0.010**
Men	83.7%	83.7%	0.991	75.6%	69.0%	**0.024**
BMI (kg/m2)	28.6 ± 4.6	28.1 ± 4.6	0.081	27.9 ± 4.5	27.5 ± 4.8	0.212
Diabetes Mellitus	28.5%	33.8%	0.064	29.7%	37.6%	**0.015**
Hypercholesterinemia	90.5%	92.0%	0.402	87.8%	89.0%	0.612
Hypertension	85.9%	88.7%	0.173	86.9%	89.4%	0.284
Smoking (current)	20.0%	17.5%	0.313	13.1%	13.5%	0.865
Left Ventricular Ejection Fraction			0.519			**0.050**
• Normal	65.6%	64.4%		73.7%	69.7%	
• Moderately reduced	19.6%	22.9%		15.0%	13.1%	
• Reduced	10.2%	8.5%		6.8%	9.1%	
• Low	4.7%	4.2%		4.5%	8.1%	
Family History of CAD	45.3%	37.8%	**0.022**	40.5%	37.2%	0.367
Heart Failure	4.3%	4.3%	0.969	4.0%	7.4%	**0.017**
NYHA Classification			0.403			**0.040**
• I	18.7%	20.8%		16.8%	12.5%	
• II	50.3%	46.3%		47.1%	41.7%	

• III	28.7%	31.7%		32.1%	40.5%	
• IV	2.3%	1.3%		3.1%	5.4%	
Prior Myocardial Infarction	38.3%	41.0%	0.381	27.1%	25.5%	0.620
Prior CABG	18.1%	24.0%	**0.014**	13.0%	19.3%	**0.008**
eGFR (ml/min/1.73^2)	74.7 ± 19.2	71.0 ± 20.1	**0.002**	70.5 ± 19.4	67.2 ± 21.9	**0.028**
Baseline Creatinine (mg/dL)	1.00 (0.89, 1.17)	1.02 (0.90, 1.20)	0.182	1.00 (0.88, 1.20)	1.00 (0.82, 1.26)	0.556

* Pericardial tamponade requiring either pericardiocentesis or surgical evacuation.

† Composite of all-cause death, myocardial infarction, stroke, target vessel-, and lesion revascularization.

MACCE, major adverse cardiac and cerebrovascular event; TLR, target lesion revascularization; TVR, target vessel revascularization.

Supplementary Table 3. Angiographic characteristics of chronic occlusive (CTO) and non-occlusive (non-CTO) percutaneous coronary intervention (PCI) with and without procedural success.

Variable	CTO PCI		P value	Non-CTO PCI		P value
	**Success (n=2489)**	**Failure (n=327)**		**Success (n=13719)**	**Failure (n=321)**	
Target vessel			**0.039**			0.270
• RCA	49.9%	41.0%		23.6%	23.7%	
• LAD	25.0%	26.3%		28.6%	32.7%	
• LM	1.0%	1.2%		7.6%	9.4%	
• CX	23.5%	30.6%		38.1%	32.7%	
• Arterial Graft	0.0%	-		0.2%	-	
• Venous Graft	0.6%	0.9%		2.0%	1.6%	
Lesion Length			0.051			0.101
• <10 mm	1.8%	3.6%		17.8%	18.2%	
• 10-20 mm	18.0%	20.3%		33.0%	38.4%	
• >20 mm	80.3%	76.1%		49.1%	43.4%	
Relevant side-branch	30.0%	26.0%	0.144	33.1%	31.8%	0.639
Calcification			**<0.001**			**<0.001**
• None	6.5%	3.7%		7.5%	3.8%	
• Mild (spots)	28.7%	15.8%		50.1%	36.7%	
• Moderate	27.9%	22.7%		27.1%	27.9%	
• Severe	36.9%	57.8%		15.3%	31.7%	

Proximal Vessel Tortuosity	19.9%	30.1%	**<0.001**	16.5%	27.4%	**<0.001**
Intra-lesion Angulation			**0.006**			**<0.001**
• None	12.1%	10.9%		11.2%	10.5%	
• Discrete (<45)	36.7%	28.8%		49.7%	40.5%	
• Moderate (45-90)	44.7%	50.3%		35.2%	38.5%	
• Severe (>90)	6.5%	10.0%		3.9%	10.5%	
Multiple Target Vessels	31.1%	35.5%	0.106	49.3%	53.3%	0.162
In-stent Restenosis	12.1%	10.8%	0.511	12.9%	10.4%	0.196
AHA/ACC Lesion Class			-			**<0.001**
• Type A	-	-		5.2%	1.9%	
• Type B1	-	-		27.1%	18.3%	
• Type B2	-	-		39.1%	37.5%	
• Type C	-	-		28.5%	42.3%	

* Pericardial tamponade requiring either pericardiocentesis or surgical evacuation.

† Composite of all-cause death, myocardial infarction, stroke, target vessel-, and lesion revascularization.

ACC, American College of Cardiology; AHA, American Heart Association; CTO, chronic total occlusion; CX, circumflex artery, LAD, left anterior descending artery; LM, left main artery; RCA, right coronary artery.

Supplementary Table 4. In-hospital and long-term complications categorized according to non-occlusive percutaneous coronary intervention (non-CTO PCI) fluoroscopy time quartiles.

**Variable**	**Overall (n=9818)**	**1st Quartile (n=2510)**	**2nd Quartile (n=2548)**	**3rd Quartile (n=2487)**	**4th Quartile (n=2273)**	**P value**
Procedural success	97.7%	98.9%	98.6%	97.8%	95.3%	**<0.001**
Technical success	99.3%	99.7%	99.6%	99.4%	99.3%	**<0.001**
Major complications *	1.78%	0.88%	1.26%	1.62%	3.56%	**<0.001**
In-hospital MACCE	1.61%	0.80%	1.18%	1.45%	3.17%	**<0.001**
• Death	0.42%	0.20%	0.27%	0.28%	0.97%	**<0.001**
• MI	0.68%	0.40%	0.39%	0.60%	1.41%	**<0.001**
• Stroke	0.17%	0.04%	0.16%	0.16%	0.35%	0.077
• TVR	1.17%	0.60%	0.71%	1.09%	2.42%	**<0.001**
• TLR	1.07%	0.48%	0.59%	1.01%	2.33%	**<0.001**
Perforation	0.64%	0.12%	0.32%	0.61%	0.64%	**<0.001**
Pericardial Tamponade †	0.21%	0.08%	0.08%	0.16%	0.57%	**<0.001**
Vascular Access Complication	1.46%	1.05%	1.32%	1.41%	2.37%	**0.013**
Bleeding	1.16%	0.60%	0.91%	1.02%	2.22%	**<0.001**
Contrast Induced Nephropathy	0.72%	0.52%	0.90%	0.80%	0.66%	0.396

30 days MACCE ‡	2.04%	1.08%	1.65%	1.89%	3.70%	**<0.001**

* Major complications are composite of in-hospital MACCE and pericardial tamponade requiring pericardiocentesis or surgical evacuation.

† Pericardial tamponade requiring either pericardiocentesis or surgical evacuation.

‡ Composite of all-cause death, myocardial infarction, stroke, target vessel-, and lesion revascularization in 30 days post-PCI.

MACCE, major adverse cardiac and cerebrovascular event; MI, myocardial infarction; TLR, target lesion revascularization; TVR, target vessel revascularization.

Supplementary Table 5. In-hospital and short-term complications categorized according to chronic occlusive percutaneous coronary intervention (CTO PCI) fluoroscopy time length quartiles.

**Variable**	**Overall (n=2720)**	**1st Quartile (n=698)**	**2nd Quartile (n=673)**	**3rd Quartile (n=669)**	**4th Quartile (n=680)**	**P value**
Procedural success	88.5%	91.3%	93.5%	87.4%	81.9%	**<0.001**
Technical success	89.7%	91.7%	94.2%	88.6%	84.4%	**<0.001**
Major complications *	2.10%	1.86%	1.78%	1.94%	2.79%	**<0.001**
In-hospital MACCE	1.36%	1.86%	1.19%	1.05%	1.32%	0.581
• Death	0.29%	0.14%	0.30%	0.15%	0.59%	0.388
• MI	0.22%	0.29%	0.15%	0.15%	0.29%	0.893
• Stroke	0.22%	0.14%	0.15%	0.15%	0.44%	0.571
• TVR	0.99%	1.43%	0.89%	0.90%	0.74%	0.580
• TLR	0.92%	1.43%	0.74%	0.75%	0.74%	0.437
Perforation	2.40%	1.00%	1.04%	2.71%	4.87%	**<0.001**
Pericardial Tamponade †	0.74%	0.00%	0.59%	0.90%	1.47%	**0.014**
Vascular Access Complication	2.05%	1.49%	1.58%	3.21%	3.27%	0.177
Bleeding	2.48%	0.72%	1.51%	2.01%	6.07%	**<0.001**
Contrast Induced Nephropathy	0.40%	0.57%	0.30%	0.30%	0.44%	0.827
30 days MACCE ‡	2.13%	2.01%	1.93%	2.54%	2.06%	0.865

* Major complications are composite of in-hospital MACCE and pericardial tamponade requiring pericardiocentesis or surgical evacuation.

† Pericardial tamponade requiring either pericardiocentesis or surgical evacuation.

‡ Composite of all-cause death, myocardial infarction, stroke, target vessel-, and lesion revascularization.

MACCE, major adverse cardiac and cerebrovascular event; MI, myocardial infarction; TLR, target lesion revascularization; TVR, target vessel revascularization.
